# ANRIL upregulates TGFBR1 to promote idiopathic pulmonary fibrosis in TGF-β1-treated lung fibroblasts via sequestering let-7d-5p

**DOI:** 10.1080/15592294.2024.2435682

**Published:** 2024-11-29

**Authors:** Weidong Wu, Nanding Yu, Weiming Chen, Yong Zhu

**Affiliations:** a Department of Thoracic Surgery, Fujian Medical University Union Hospital, Fuzhou, Fujian, China; b Fujian Key Laboratory of Cardio-Thoracic Surgery, Fujian Medical University, Fuzhou, Fujian, China; c Department of Pulmonary and Critical Care Medicine, Fujian Medical University Union Hospital, Fuzhou, Fujian, China; d Department of Geriatric Medicine, Fujian Medical University Union Hospital, Fuzhou, Fujian, China

**Keywords:** Idiopathic pulmonary fibrosis, ANRIL, let-7d-5p, TGFBR1

## Abstract

Idiopathic pulmonary fibrosis (IPF) is a progressive and life-threatening respiratory disease characterized by worsening lung function due to excessive scarring. The objective of this study was to investigate the role of the long non-coding RNA ANRIL (antisense non-coding RNA in the INK4 locus) in the development of IPF. Our research revealed a significant increase in ANRIL expression in pulmonary fibrosis, consistent with prior studies indicating elevated ANRIL levels in fibrotic tissues. *In vitro* experiments demonstrated that elevated ANRIL expression promoted fibroblast activation, as evidenced by the upregulation of fibrosis-related markers. Mechanistically, we found that ANRIL interacts with let-7d-5p, a microRNA involved in gene regulation, acting as a sponge for let-7d-5p. Functional experiments confirmed a potential influence of let-7d-5p on fibroblast activation through direct interaction with ANRIL. Furthermore, our investigation identified TGFBR1 as a potential mediator of ANRIL’s fibrogenic effects. Silence of TGFBR1 mitigated the fibrotic phenotype induced by ANRIL overexpression. Collectively, these results suggest that ANRIL promotes fibroblast activation and fibrosis development, possibly through the let-7d-5p/TGFBR1 axis, indicating that ANRIL could be a potential therapeutic target for pulmonary fibrosis.

## Introduction

IPF is a chronic, progressive lung disease characterized by fibrosis of uncertain etiology. The typical pathological manifestation is often observed as usual interstitial pneumonitis (UIP), which manifests as widespread inflammation and structural damage to the air sacs, ultimately leading to pulmonary interstitial fibrosis [1]. Chronic and progressive dyspnea, interstitial lung infiltration, decreased lung compliance, and impaired gas exchange are the main clinical features [2,3]. The prevalence of IPF is increasing annually, leading to higher rates of disability and mortality, with a grim prognosis. However, there is currently no effective treatment available for IPF [1,4,5]. Patients diagnosed with IPF typically have an average survival period ranging from 2.5 to 3.5 years, with a noticeable decrease in survival rates over time [6]. The 3-year survival rate is 50%, while the 5-year survival rate is only 20%. Additionally, recent cases of COVID-19 have shown consistent changes in the pulmonary interstitium on CT scans. Examination of deceased individuals revealed mucus accumulation in the lung interstitium along with visible fibrous strands. Older individuals are at higher risk of COVID-19, and the likelihood of developing pulmonary fibrosis post-treatment increases, adding strain to public healthcare finances in the future.

Currently, the main medications used for treating IPF are pirfenidone and nintedanib. Although these drugs can only delay the progression of the disease, they are unable to effectively prevent its onset and advancement [7]. Lung transplantation is the only effective treatment method available in clinical practice. In 2015, the International Society for Heart and Lung Transplantation reported over 51,440 lung transplant procedures worldwide. Survival rates post-transplant were recorded as 89% at 3 months, 80% at 1 year, 65% at 3 years, 54% at 5 years, and 31% at 10 years [8]. However, the limited availability of donor lungs and the risk of immune rejection make lung transplantation unattainable for many patients. With China’s aging population and the subsequent increase in elderly individuals, IPF is expected to become a significant public health concern, causing substantial economic and social burdens on patients, families, and society as a whole. Therefore, actively seeking new and effective treatment methods and targets is crucial.

Long non-coding RNAs (lncRNAs) are a group of non-coding RNAs exceeding 200 nucleotides in length that play significant regulatory roles in various cellular processes [9–11]. Recent studies have linked lncRNAs to the progression of fibrosis, particularly in idiopathic pulmonary fibrosis (IPF). These lncRNAs represent potential therapeutic targets capable of modulating gene expression and signaling pathways involved in fibrotic processes [12–14]. One noteworthy example is ANRIL (antisense non-coding RNA in the INK4 locus), which has been extensively studied in contexts like cancer and cardiovascular diseases [15–17]. Research has indicated that the expression of lncRNA LINC01140 is elevated in lung biopsies from IPF patients and in fibroblasts derived from these individuals. Functional knockdown studies reveal that LINC01140 promotes proliferation in both control and IPF-derived fibroblasts. Additionally, the absence of LINC01140 is linked to heightened inflammatory responses, which are more pronounced in IPF fibroblasts compared to controls [18]. Conversely, recent findings show that lncRNA SNHG1 (lnc-SNHG1) is overexpressed in fibrotic lung tissues of murine models and in TGF-β1-treated fibroblasts. Manipulating lnc-SNHG1 expression has demonstrated that its high levels enhance fibroblast migration, invasion, and fibrosis-related molecule secretion, while lower levels produce the opposite effects [19]. These results suggest that lncRNAs could significantly influence the cellular and molecular mechanisms involved in IPF development. Further research is needed to explore these pathways and better understand IPF pathogenesis. Understanding the function of ANRIL in fibrosis could provide valuable insights into the pathogenesis of IPF and potentially lead to novel treatment strategies.

This study aims to investigate the involvement of ANRIL in fibrosis, specifically in relation to IPF. We will assess its expression levels and explore its potential correlation with the severity of fibrosis. Moreover, we will elucidate the molecular mechanisms through which ANRIL may contribute to fibrosis, including its interactions with key signaling pathways and target genes associated with fibrogenesis. Our goal is to deepen our understanding of fibrosis by uncovering the roles of ANRIL and identifying potential targets for therapeutic interventions.

## Methods and materials

### Mouse model

Male C57BL/6 mice, between the ages of 6 and 8 weeks, and weighing an average of 18–23 g, were utilized. An experimental model of pulmonary fibrosis was created in mice by administering BLM (3 mg/kg, Selleck) directly into the trachea while under anesthesia. This led to the development of pulmonary fibrosis 28 days later. Saline was administered to the control mice. Each experimental group was assigned animals randomly. The assessors were not aware of the group assignment. The ethical committee of Fujian Medical University Union Hospital approved all animal experiments.

### Cell culture

The MRC-5 cell line, derived from human fetal lung fibroblasts, was commercially acquired from the Cell Bank of Chinese Academy of Sciences. The cells were cultured at 37°C in MEM medium supplemented with 10% FBS and 5% CO_2_.The excessive growth and stimulation of typical human lung fibroblasts were conducted following the methods described earlier. In summary, MRC-5 cells underwent a 48-hour period of starvation before being exposed to fetal calf serum (FCS) at concentrations of either 2% or 5%. Additionally, they were treated with PDGF-BB at doses of either 30 or 60 ng/ml, and IGF-1 at concentrations of either 100 or 200 ng/ml, for a duration of 48 hours to stimulate the proliferation of fibroblasts. Subsequently, the cells were incubated with TGF-β1 at concentrations of either 5 or 10 ng/ml for 48 hours to induce fibroblast activation. Solarbio (China) was the source of all growth factors and cytokines.

### Cell transfection

MRC-5 cells were transfected temporarily with ANRIL/TGFBR1 plasmid or ANRIL/TGFBR1 small interference RNA (siRNA) using Lipofectamine 2000 (Invitrogen, USA) as per the instructions provided by the manufacturer. Ribobio (Guangzhou, China) synthesized both siRNA and a siNC, which served as a negative control. Lipofectamine 2000 (Invitrogen, USA) was used to deliver microRNA inhibitors (let-7d-5p inhibitor and let-7d-5p inhibitor) or their corresponding negative controls (NC mimics and NC inhibitor).

### Real-time quantitative reverse transcription polymerase chain reaction

TRIzol reagent was used to extract total RNA from the lung tissues and MRC-5 cells. The NanoDrop 8000 (Thermo) was used to assess the RNA’s concentration and purity. Total RNA was reverse transcribed using random primers with cDNA reverse transcription kits from TransGene Biotech in China. SYBR Green was used to quantify the mRNA on the ABI 7500 Fast Real-time PCR system from Applied Biosystems in the United States. Normalization was performed using the GAPDH gene. The efficiency of the primer was evaluated through a range of dilutions, and the specificity of the primer was determined by analyzing the dissociation curve. The sequences of primers can be found in Table S1 of the Supporting Information. The analysis of the data was conducted using the 2^−ΔΔCT^ technique.

### Cell proliferation assays

MRC-5 lung fibroblasts were cultured in 24-well plates to evaluate their cellular proliferation. The Cell-Light EdU DNA Cell Proliferation Kit (RiboBio, Guangzhou, China) was utilized to assess the proliferation of fibroblasts. All the experimental procedures followed the guidelines provided by the manufacturer.

### Scratch wound-healing assay

An *in vitro* scratch assay was conducted to assess the migratory ability of lung fibroblasts as previous described [13]. The cells were placed on 6-well dishes and kept under standard cultural conditions for incubation. During the process of cell confluence, simulated wounds were created. Additionally, the cells were transfected either with or without alterations. The Nikon TS100 microscope (Nikon, Tokyo, Japan) was utilized to observe the phase-microscopy images at various time intervals.

### Immunofluorescence staining

After being fixed in 4% PFA for 30 minutes at room temperature, lung fibroblasts underwent permeabilization and blocking treatment. The cells were cultured with antibodies targeting a-SMA (1:100, Abcam) for an extended period at a temperature of 4°C. The following day, the cells were rinsed with PBS three times and subsequently exposed to fluorescein isothiocyanate (FITC)-labeled goat anti-mouse antibodies under dark conditions. DAPI (Roche Molecular Biochemicals) was used to stain the nuclei for a duration of 5 minutes. The immunofluorescence was examined using a fluorescence microscope from Nikon 80i in Tokyo, Japan.

### Luciferase Reporter assay

General Biosystems (Anhui) Co. Ltd. synthesized and PCR amplified the ANRIL 3’ UTR and TGFBR1 3’ UTR, which include the let-7d-5p binding sites that are conserved. Using the QuikChange II XL Site-Directed Mutagenesis Kit (Stratagene), we created mutations in the ANRIL 3’ UTR and TGFBR1 3’ UTR that do not have let-7d-5p binding sites. The wild type (WT) and mutated 3’ UTR sequences were inserted into the pGL3 vector (Promega) right after the luciferase gene’s coding region. Then, the luciferase vector (0.1 mg) was co-transfected into HEK293 cells with let-7d-5p mimics/inhibitors or 3’ UTR using Lipofectamine 2000 (Invitrogen, Carlsbad, CA, USA). In order to maintain internal control, a renilla luciferase reporter of 10 ng was additionally incorporated. Following a 48-hour transfection, the cells were gathered and the dual-luciferase activities were assessed using a luminometer as per the guidelines provided by the manufacturer (Promega, Fitchburg, WI, USA).

### RNA-binding protein immunoprecipitation (RIP)

The RNA-Binding Protein Immunoprecipitation Kit from MagnaRIP^TM^ (Millipore, USA) was utilized to conduct RNA-binding protein immunoprecipitation (RIP) as per the guidelines provided by the manufacturer as previously described [20]. In short, RIP assays were conducted using AGO2 antibody (Abcam) or a negative control immunoglobulin G (IgG). The cells were treated with TGF-β1 for 48 hours, then collected and lysed using a buffer specifically designed for RNA extraction. Next, 5 milligrams of AGO2 antibody or IgG were mixed with 50 milliliters of magnetic bead suspension and incubated at room temperature for 30 minutes to prepare the beads for immunoprecipitation. The magnetic beads were suspended again using RIP immunoprecipitation buffer and left to incubate with the cells overnight at a temperature of 4°C. On the following day, the RNA/magnetic bead combination was rinsed and suspended in proteinase K solution for the purpose of protein isolation. After immunoprecipitation, the RNA was subsequently purified for analysis using agarose gel electrophoresis or qRT-PCR. Negative controls were provided by IgG enrichment.

### Western blot

Protein samples were obtained from cells and tissues by utilizing RIPA lysis buffer containing protease inhibitor (Beyotime, Jiangsu, China). The proteins were isolated using a 10% or 8% SDS polyacrylamide gel and then transferred to a nitrocellulose membrane (Pall Life Sciences, Ann Arbor, MI, USA). Antibodies against TGFBR1 (1:1000, Proteintech 30,117–1-AP), Acta2 (1:2000, CST, #14968), FN1 (1:2000, CST, #26836), Col1a1 (1:2000, CST, #72026), Col3a1(1:2000, CST, #30565) were used to detect proteins. To serve as a loading control, GAPDH (1:2000, Proteintech 14,395–1-AP) was utilized. After incubating the membranes with secondary antibodies, they were kept at room temperature for 1 hour. Protein expression was detected and quantified using Odyssey (Odyssey CLX, Biosciences, USA) in the end.

### Statistics and analysis

The data is shown as the average ± SEM of at least three separate trials. To determine statistical significance, either a two-tailed t-test or a one-way analysis of variance (ANOVA) with a post-test Bonferroni corrected t-test was employed. A difference was considered statistically significant if *p* < 0.05. GraphPad Prism 8.0 was utilized for conducting the statistical analyses.

## Results

### ANRIL was upregulated in IPF lung fibroblasts

**Figure 1.**
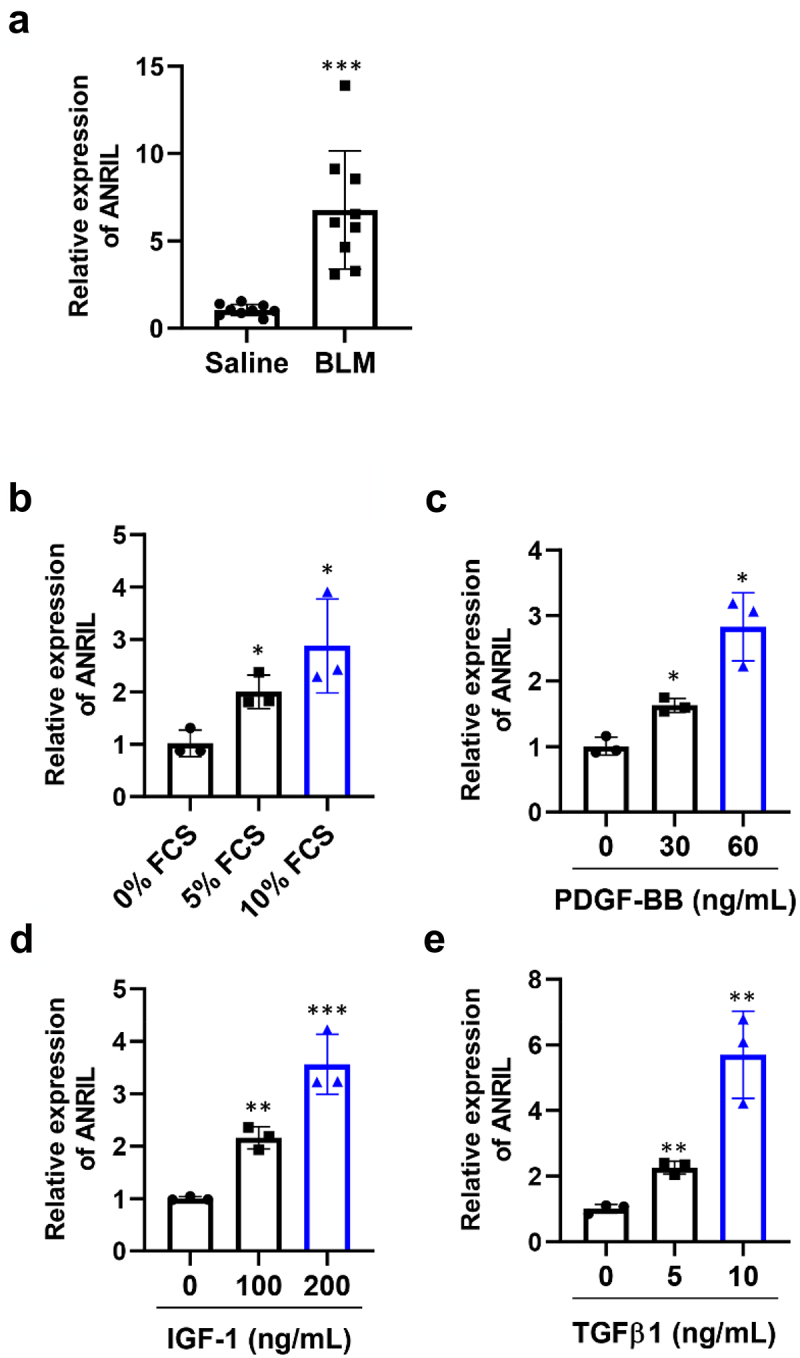
ANRIL was upregulated in IPF lung fibroblasts. (a). qRT-pcr analysis of the expression of ANRIL in the lungs of blm-treated mice, *n* = 6. (b–e). MRC-5 cells were stimulated with fetal calf serum (FCS; 2% or 5%), platelet-derived growth factor-bb (PDGF-BB; 30 or 60 ng/ml), insulin-like growth factor 1 (IGF-1; 100 or 200 ng/ml), and transforming growth factor-β1 (TGF-β1; 5 or 10 ng/ml) for 6 h, respectively. ANRIL expression was measured by qRT-pcr, *n* = 3. Data are presented as mean ± SEM; *p < 0.05, **p < 0.01, ***p < 0.001.

Increasing evidence indicated the participation of lncRNAs in the progression of organ fibrosis [14,21,22]. In order to determine the impact of ANRIL on lung fibrosis, we created an animal model by administering BLM through intratracheal injection. After a period of four weeks, it was discovered that the BLM-injected mice exhibited a significant increase in ANRIL levels (Figure 1a). Therefore, we had a suspicion that ANRIL played a role in pulmonary fibrosis. Consistent with the findings obtained in live organisms, we observed similar outcomes when growth factors and cytokines (FCS, for example) were introduced. The growth factors and cytokines (PDGF-BB, IGF-1, and TGF-β1) that have been known to promote the proliferation and activation of lung fibroblasts in laboratory conditions were added to the culture medium. As a result, the expression of ANRIL increased in a dose-dependent manner, indicating that the activation of fibroblasts led to a gradual increase in ANRIL expression (Figure 1b–1e).

### ANRIL contribute to fibrogenesis in cultured lung fibroblasts

**Figure 2.**
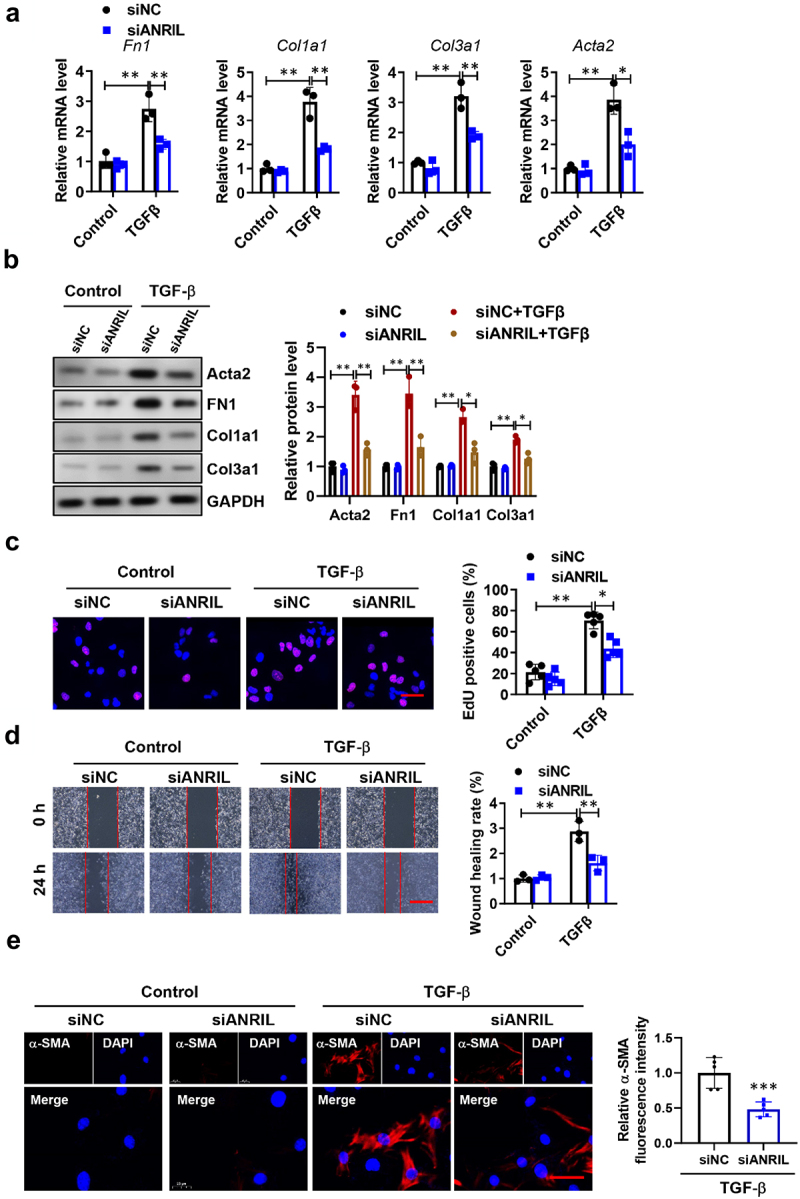
Knockdown of ANRIL inhibited TGF-b induced fibroblasts proliferation and activation. (a) The relative mRNA levels of *Fn1*, *Col1a1*, *Col3a1*, and *Acta2* in ANRIL silenced MRC-5 cells after TGF-b induction; *n* = 3. (b) The relative protein levels of Fn1, Col1a1, Col3a1, and Acta2 in ANRIL silenced MRC-5 cells after TGF-β induction; *n* = 3. (c) EdU staining demonstrated the effect of ANRIL knockdown on lung fibroblast proliferation. Scale bar, 50 mm, *n* = 5. (d) Scratch assay for the evaluation of migration of lung fibroblasts after ANRIL knockdown. Scale bar, 200 mm, *n* = 5. (e) Immunofluorescence staining indicated that TGF-β1-induced a-sma positive cells were impeded by the reduced expression of ANRIL in MRC-5 fibroblasts. Scale bar, 50 mm, *n* = 5. Data are presented as mean ± SEM; *p < 0.05, **p < 0.01, ***p < 0.001.

To explore whether ANRIL played a potential role in IPF progression, we silenced ANRIL expression in MRC-5 cells using siRNA transfection (Figure S1A). mRNA and protein levels of Fn1, Col1a1, Col3a1, and Acta2 in fibroblasts significantly induced by TGF-β1, whereas these effects were abrogated by knockdown of ANRIL (Figure 2a,b). Given that the activation of fibroblasts plays a crucial role in pulmonary fibrosis, we assessed the impact of ANRIL on the functioning of lung fibroblasts. According to EdU fluorescence staining (Figure 2c), it was confirmed that ANRIL silence effectively inhibited the enhanced cell proliferation caused by TGF-β1. Figure 2d demonstrated that the migratory capacity of lung fibroblasts was hindered by the suppression of ANRIL in a wound healing experiment. In the presence of ANRIL knockdown, the presence of α-SMA positive myofibroblasts was reduced as observed in the immunofluorescence staining, indicating a decrease in the transition of fibroblasts into myofibroblasts (Figure 2e).

### ANRIL served as a sponge for let-7d-5p in MRC-5 fibroblasts

**Figure 3.**
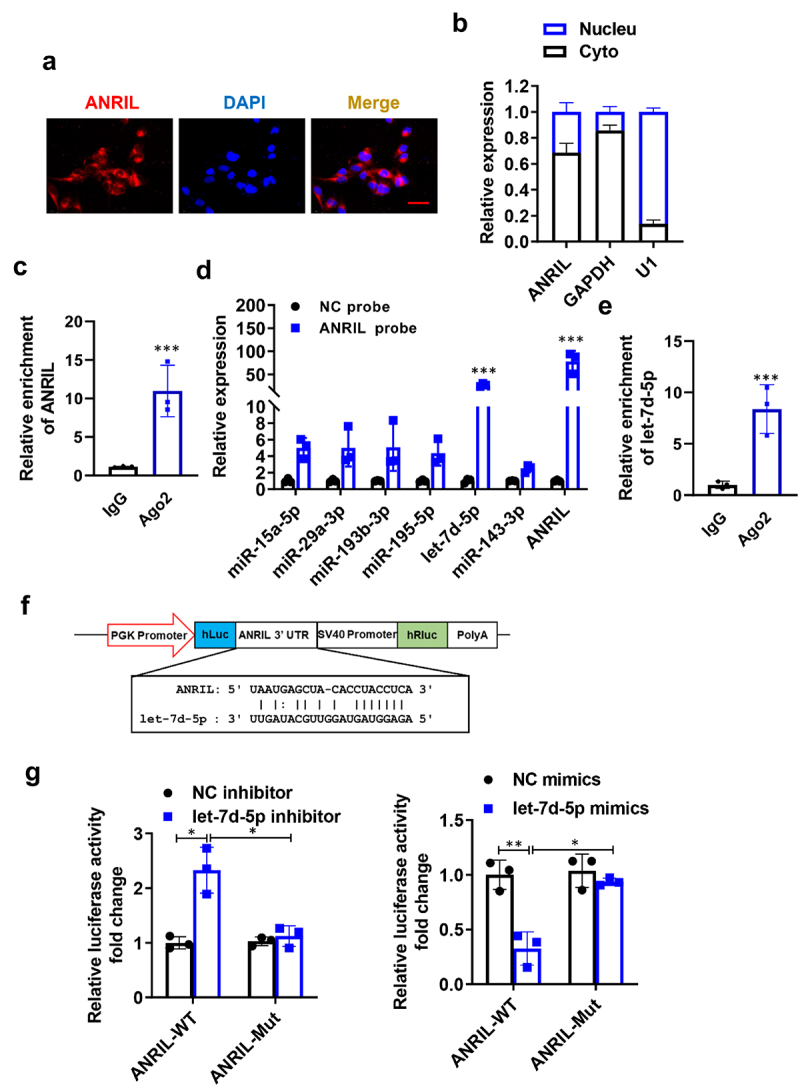
ANRIL serves as a sponge for let-7d-5p in MRC-5 fibroblasts. (a) Representative FISH images showing the cellular localization of ANRIL. The ANRIL probe was labeled with Cy3 (red), and the nuclei were stained with DAPI (blue). Scale bar = 50 μm. (b) qRT-qPCR analysis following subcellular fractionation of ANRIL, *n* = 5. (c) ANRIL interacted with Ago2 in MRC-5 cells, *n* = 3. (d) RNA pull-down assay was carried out using an ANRIL probe, *n* = 5. E. let-7d-5p interacted with Ago2 in MRC-5 cells, *n* = 3. F. Predicted binding sites of ANRIL and let-7d-5p. (g) luciferase activity of ANRIL-WT and ANRIL-Mut in MRC-5 co-transfected with let-7d-5p inhibitor or mimics, *n* = 3. Data are presented as mean ± SEM; *p < 0.05, **p < 0.01, ***p < 0.001.

The mechanism of action of lncRNAs is mainly determined by their cellular localization [23]. Hence, experiments were carried out involving the separation of subcellular components and the use of fluorescence in situ hybridization (FISH) assays. Upon investigation, it was discovered that ANRIL was present in both the cytoplasm and nucleus, with a predominant localization in the cytoplasm during normal physiological conditions (Figure 3a,3b). Recent studies have clearly shown that long non-coding RNAs (lncRNAs) function as microRNA (miRNA) sponges in various diseases, including idiopathic pulmonary fibrosis (IPF). Initially, we conducted a RIP assay on AGO2, which had diverse functions in the miRNA pathway and contributed to the formation of pre-miRNA by generating pre-miRNA [24,25]. In MRC-5 cells (Figure 3c), we discovered a strong interaction between AGO2 and ANRIL. Our objective was to identify the miRNA that could directly bind to ANRIL. To accomplish this, we employed an ANRIL probe to screen the interaction between ANRIL and multiple predicted microRNAs. According to the findings in Figure 3d, ANRIL exhibited the highest ability to decrease the levels of let-7d-5p compared to the other options. In MRC-5 cells, the RIP assay provided additional evidence that AGO2 had a strong interaction with let-7d-5p (Figure 3e).

Moreover, the bioinformatics analysis indicated that the 3’UTR region of ANRIL contained a targeting motif for let-7d-5p. Consequently, we developed a luciferase assay system to examine the impact of let-7d-5p on the regulation of ANRIL. In this experiment, the firefly luciferase cassette was connected to either the normal or altered ANRIL 3’UTR sections, where the altered ANRIL 3’UTR lacked the ability to bind with let-7d-5p (Figure 3f). When MRC-5 cells were transfected with the luciferase plasmids, the intensity of luciferase carrying the wild type region was reduced compared to the mutated region (Figure 3g) when let-7d-5p was simultaneously overexpressed. In addition, we employed a let-7d-5p inhibitor to examine if the luciferase expression was restored, and the outcome indicated an upregulation in the wild-type expression (Figure 3g). Above all, let-7d-5p directly binds ANRIL and affects its stability in MRC-5 fibroblasts.

### Role of ANRIL as a regulator for let-7d-5p in promoting fibroblasts proliferation and activation

**Figure 4.**
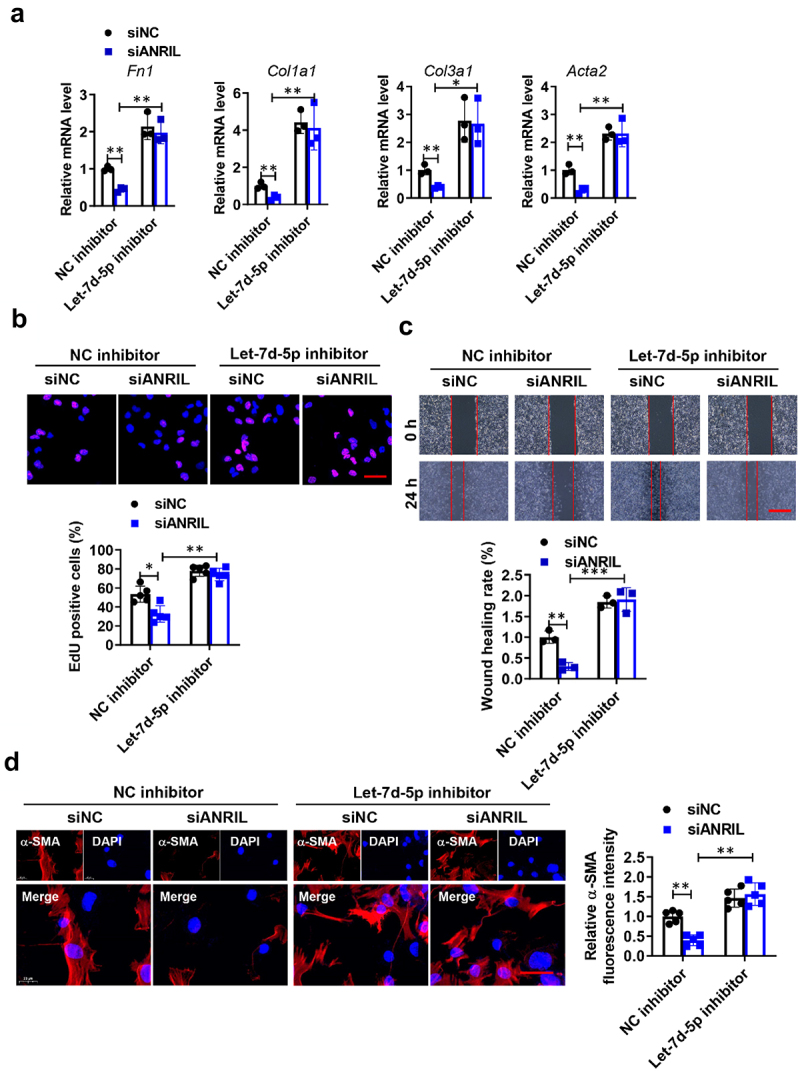
Silencing of let-7d-5p abolishes the anti-fibrotic effect of ANRIL knockdown. (a) qRT-pcr results indicate that the let-7d-5p inhibitor normalized the inhibitory effect of ANRIL knockdown on the mRNAs of fibrosis-related genes, *n* = 3. (b) EdU results (scale bar, 50 mm; *n* = 5) and (c) wound healing assays show the proliferation and migration of MRC-5 fibroblasts after different treatments, scale bar, 50 mm, *n* = 5. (d) Immunofluorescence analysis shows that different treatments on the transition of fibroblasts into myofibroblasts, *n* = 5. Data are presented as mean ± SEM; *p < 0.05, **p < 0.01, ***p < 0.001.

After a period of four weeks, it was discovered that the BLM-injected mice exhibited a significant decrease in let-7d-5p levels (Figure S2A). Moreover, the expression of let-7d-5p decreased in a dose-dependent manner with treatment of growth factors and cytokines (PDGF-BB, IGF-1, and TGF-β1) (Figure S2B-E). Subsequently, we explored the biological function of the ANRIL/let-7d-5p pathway in the activation and proliferation of lung fibroblasts. To investigate the impact of ANRIL on fibrogenesis and explore the potential involvement of let-7d-5p, siRNA targeting ANRIL was introduced into lung fibroblasts. The activation of fibroblasts induced by TGF-β1 was inhibited when ANRIL was suppressed. However, when let-7d-5p expression was increased, the inhibitory effects were nullified. Conversely, the inhibitory effects were successfully counteracted when the let-7d-5p inhibitor was expressed simultaneously (Figure 4 and Figure S3). Together, we showed that the long non-coding RNA ANRIL plays a crucial role in activating lung fibroblasts by acting as a reservoir for let-7d-5p.

### ANRIL requires TGFBR1 for its pro-fibrotic effects

**Figure 5.**
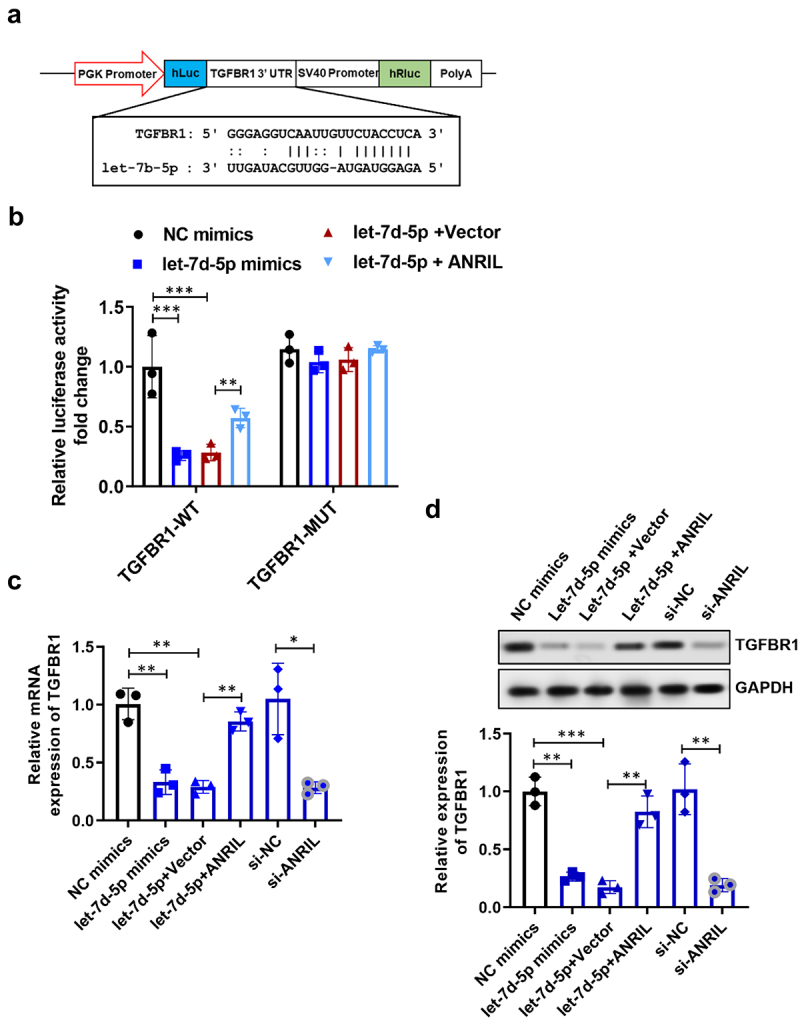
ANRIL regulated TGFBR1 expression by interacting with let-7d-5p. (a) Predicted binding sites of TGFBR1 and let-7d-5p. (b) The luciferase assay of TGFBR1 3’UTR in MRC-5 cells co-transfected with let-7d-5p mimics and siRNA for ANRIL, *n* = 3. (c) The mRNA level of TGFBR1 in MRC-5 cells co-transfected with let-7d-5p mimics and siRNA for ANRIL, *n* = 5. (d) The protein expression of TGFBR1 in MRC-5 cells co-transfected with let-7d-5p mimics and siRNA for ANRIL, *n* = 3. Data are presented as mean ± SEM; *p < 0.05, **p < 0.01, ***p < 0.001.

In order to uncover the underlying mechanisms that mediate the anti-fibrotic function of let-7d-5p, we utilized the TargetScan database to detect potential targets of let-7d-5p. The let-7d-5p had a complementary base sequence in the TGFBR1 3’ UTR, as depicted in Figure 5a. Subsequently, we created a luciferase reporter vector that contained a let-7d-5p binding site targeting fragment (TGFBR1-WT) as well as a mutant sequence (TGFBR1-MUT). The luciferase reporter assay showed that let-7d-5p inhibited the fluorescence activity of the TGFBR1-WT luciferase vector. However, there was no direct relationship between let-7d-5p and the TGFBR1-MUT vector. Interestingly, co-transfection with ANRIL was able to restore the luciferase activity in the TGFBR1-WT group (Figure 5b). Furthermore, the excessive presence of let-7d-5p suppressed the expression of TGFBR1 at both the protein and mRNA levels. However, the co-expression of ANRIL was able to revive TGFBR1 expression, as depicted in Figure 5c,5d.

**Figure 6.**
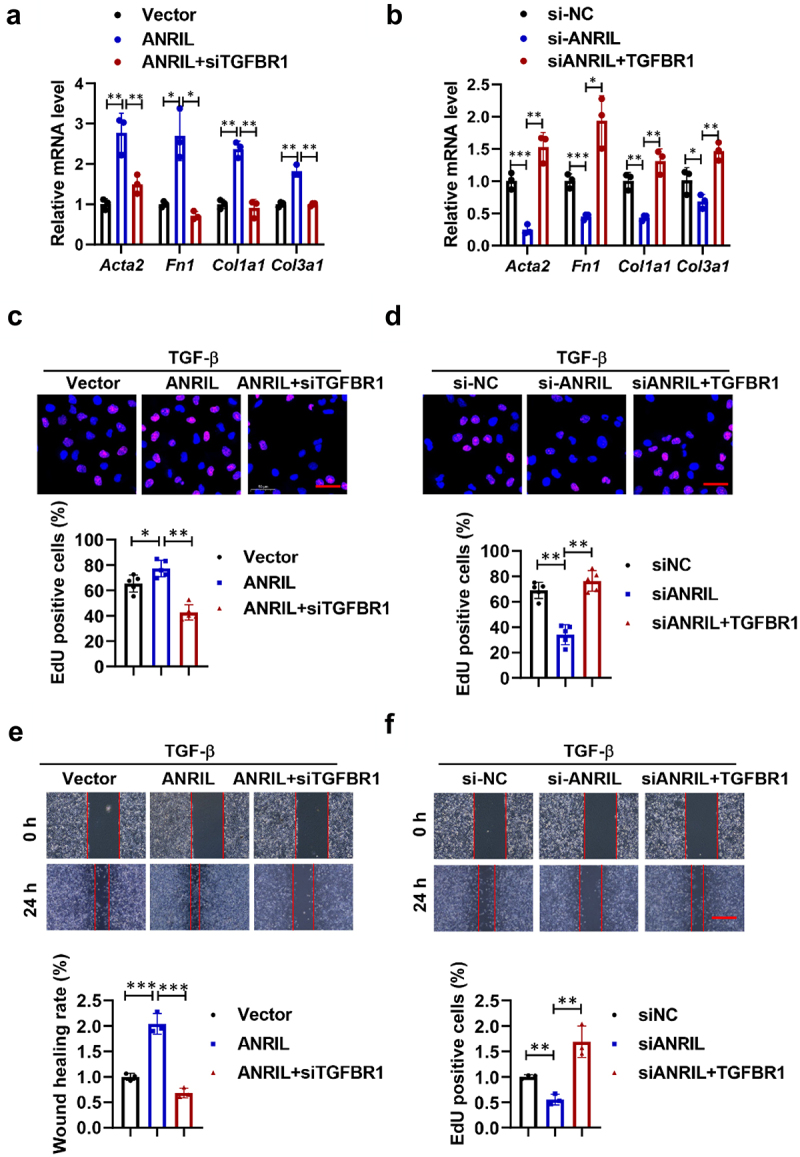
ANRIL promotes lung fibroblasts activation via TGFBR1. (a,b). The relative mRNA levels of *Fn1*, *Col1a1*, *Col3a1*, and *Acta2* in MRC-5 cells with different treatments after TGF-b induction, *n* = 3. (c,d). EdU results show the proliferation and migration of MRC-5 fibroblasts after different treatments, scale bar, 50 mm, *n* = 5. (e,f). wound healing assays show the proliferation and migration of MRC-5 fibroblasts after different treatments, scale bar, 50 mm, *n* = 5. Data are presented as mean ± SEM; *p < 0.05, **p < 0.01, ***p < 0.001.

We next examined whether ANRIL’s profibrotic effect is mediated by TGFBR1. Increased ANRIL expression elevated both the mRNA (Figure 6a) and protein levels (Figure S4A) of Fn1, Col1a1, Col3a1, and Acta2 in TGF-β1-treated fibroblasts. However, these increases were significantly reduced following TGFBR1 knockdown. Conversely, overexpressing TGFBR1 reversed the suppression of these genes induced by ANRIL knockdown. Additionally, ANRIL overexpression promoted proliferation, migration and fibroblast-myofibroblast transition in lung fibroblasts, effects that were mitigated by TGFBR1 knockdown. Furthermore, TGFBR1 overexpression counteracted the enhanced fibroblast activation associated with ANRIL overexpression (Figure 6c–f and Figure S4B). These results suggest that ANRIL drives lung fibroblast activation via the let-7d-5p/TGFBR1 pathway.

## Discussion

Fibroblast activation plays a crucial role in the progression and development of fibrotic diseases, marked by excessive accumulation of extracellular matrix (ECM) components. In this study, we identified an increase in ANRIL expression in mice treated with BLM and in lung fibroblasts exposed to growth factors and cytokines. Our findings reveal that ANRIL modulates TGFBR1 expression by binding to let-7d-5p, which in turn promotes fibroblast activation and ECM deposition. This insight highlights ANRIL as a potential target for IPF prevention and treatment.

Recent advancements in research have increasingly highlighted the molecular mechanisms underlying IPF [26], with long noncoding RNAs (lncRNAs) emerging as a significant area of interest. LncRNAs are recognized as crucial regulators in various diseases, and their dysregulated expression is linked to fibrosis, including IPF [27]. Recent findings indicate that activating transcription factor 3 (ATF3) can stimulate LINC00941/lncIAPF to promote the differentiation of fibroblasts into myoblasts by inhibiting ELAVL1/HuR-dependent autophagy in pulmonary fibrosis [14]. Additionally, silencing lncRNA SNHG6 has been shown to reduce bleomycin-induced pulmonary fibrosis in mice through the miR-26a-5p/TGF-β1-smads pathway [28]. These studies underscore the significant role of lncRNAs in IPF and their potential impact on future diagnostic and therapeutic strategies. In the context of pulmonary fibrosis, we observed a significant rise in ANRIL expression, consistent with previous studies that reported heightened ANRIL levels in fibrotic tissues [29–31]. This finding implies ANRIL’s potential role in the development of fibrosis, as reported in previous studies [29,32]. Furthermore, our *in vitro* studies revealed that the overexpression of ANRIL resulted in increased activation of fibroblasts. This was evident from the upregulation of fibrosis markers, which are crucial in the remodeling of the extracellular matrix and the transformation of fibroblasts into myofibroblasts [33,34]. Furthermore, the overexpression of ANRIL also stimulated the proliferation and invasion of lung fibroblasts. Our study unveils, for the first time, the upregulation of ANRIL in the lung tissues of mice treated with BLM, leading to the enhancement of fibroblast proliferation and differentiation, exacerbating pulmonary fibrosis.

Studies have shown that lncRNAs with target sequences resembling those of miRNAs can modulate RNA levels by sequestering miRNAs or by directly interacting with them to influence their activity. While both miRNAs and lncRNAs are crucial in the development and progression of IPF, their specific regulatory networks in this context are not yet fully understood. Consequently, extensive research [35–37] has focused on exploring the intrinsic regulatory interactions between lncRNAs and miRNAs in IPF pathogenesis. To gain further insight into the relationship between ANRIL and fibroblast activation, we explored the potential interaction of ANRIL with let-7d-5p and TGFBR1. Our study results revealed that ANRIL can bind to let-7d-5p, a microRNA involved in various cellular processes. Subcellular fractionation and in situ hybridization experiments confirmed the cytoplasmic localization of ANRIL in MRC-5 fibroblasts. Additionally, ANRIL interacts with AGO2, let-7d-5p, and other proteins involved in miRNA-mediated gene regulation. Bioinformatics analysis identified possible let-7d-5p binding sites in the 3’ UTR region of ANRIL. Functional assays demonstrated direct interaction between let-7d-5p and ANRIL, affecting its stability and potentially modulating fibroblast activation. The identification of functional domains within ANRIL addresses the challenge posed by its lengthy nucleotide sequences, thereby enhancing its potential as a targeted therapeutic agent for treating pulmonary fibrosis.

Furthermore, our study shed light on the involvement of TGFBR1 in the regulatory network linking ANRIL to fibroblast activation. Acting as a receptor for TGF-β, TGFBR1 plays a crucial role in transmitting signals through the TGF-β pathway, impacting cellular growth and ECM production [38–40]. Through bioinformatics analysis and functional experiments, we uncovered TGFBR1 as a target of let-7d-5p, suggesting a potential mechanism by which ANRIL exerts its fibrogenic effects. Importantly, inhibition of TGFBR1 reversed the fibrotic traits induced by ANRIL overexpression, indicating a beneficial role of TGFBR1 in fibroblast activation. While our research provides valuable insights into the intricate interplay among ANRIL, let-7d-5p, and TGFBR1 in fibroblast activation and fibrogenesis, it is essential to acknowledge certain limitations. To validate these findings, further investigations using in vivo models and human primary fibroblasts are warranted, as our study primarily focused on in vitro experiments with cell lines. Additionally, despite presenting evidence supporting the involvement of ANRIL, let-7d-5p, and TGFBR1 in fibroblast activation, additional research is needed to elucidate the precise molecular mechanisms and signaling pathways involved.

## Conclusion

In summary, our study reveals a key link between ANRIL and TGFBR1 in fibroblast activation and fibrosis. The increased ANRIL expression in fibrotic tissues suggests its role in fibrosis development. In vitro experiments show that ANRIL overexpression enhances fibroblast activation, proliferation, and invasion, and interacts with let-7d-5p while being located in the fibroblast cytoplasm. TGFBR1 is identified as a central component in the ANRIL-let-7d-5p-fibroblast activation pathway. Targeting TGFBR1 could counteract the fibrotic effects of ANRIL overexpression, highlighting its potential for therapeutic interventions. Further research with in vivo models and human fibroblasts is needed to confirm these findings and explore the underlying molecular mechanisms. Despite these limitations, our research offers valuable insights into the ANRIL-let-7d-5p-TGFBR1 axis, suggesting promising targets for treating fibrotic disorders and improving patient outcomes.

## Authors’ contributions

Weidong Wu conducted animal experiments. Nanding Yu performed the in vitro experiments. Weidong Wu, Nanding Yu, Weiming Chen, and Yong Zhu analyzed the data and wrote the manuscript. Yong Zhu is the guarantor of this work and, as such, had full access to all the data in the study and takes responsibility for the integrity of the data and the accuracy of the data analysis.

## Supplementary Material

-) Supplementary Table 1.docx

original_images_of_western_blot.docx

Supplementary figure.pdf

## Data Availability

The datasets used and/or analyzed in this study are available from the corresponding author on reasonable request.
