# Supplementary material for: ANRIL upregulates TGFBR1 to promote idiopathic pulmonary fibrosis in TGF-β1-treated lung fibroblasts via sequestering let-7d-5p
Source: Epigenetics. 2024 Nov 29;19(1):2435682. doi: 10.1080/15592294.2024.2435682 (PMC11610569; doi:10.1080/15592294.2024.2435682)
Supplement: -) Supplementary Table 1.docx [file KEPI_A_2435682_SM9171.docx]

| Gene | Forward (5´-3´) | Reverse (5´-3´) |
| --- | --- | --- |
| *Fn1* | CGGTGGCTGTCAGTCAAAG | AAACCTCGGCTTCCTCCATAA |
| *Col1a1* | GAGGGCCAAGACGAAGACATC | CAGATCACGTCATCGCACAAC |
| *Col3a1* | GGAGCTGGCTACTTCTCGC | GGGAACATCCTCCTTCAACAG |
| *Acta2* | AAAAGACAGCTACGTGGGTGA | GCCATGTTCTATCGGGTACTTC |
| ANRIL | CTCTCATCTGATCTCCGTCCT | TCACATCCAAGACAGCAAGT |
| GAPDH | TGTGGGCATCAATGGATTTGG | ACACCATGTATTCCGGGTCAAT |

**Supplementary Table 1：Primers used for qPCR assay**
