# Supplementary figures and images for: ANRIL upregulates TGFBR1 to promote idiopathic pulmonary fibrosis in TGF-β1-treated lung fibroblasts via sequestering let-7d-5p
Source: Epigenetics. 2024 Nov 29;19(1):2435682. doi: 10.1080/15592294.2024.2435682 (PMC11610569; doi:10.1080/15592294.2024.2435682)

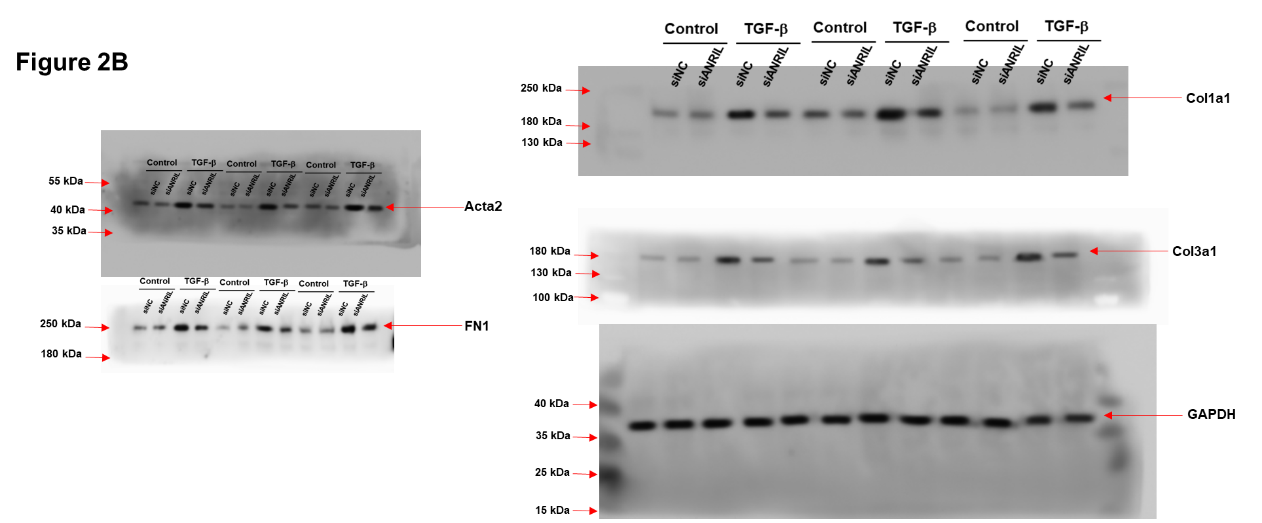


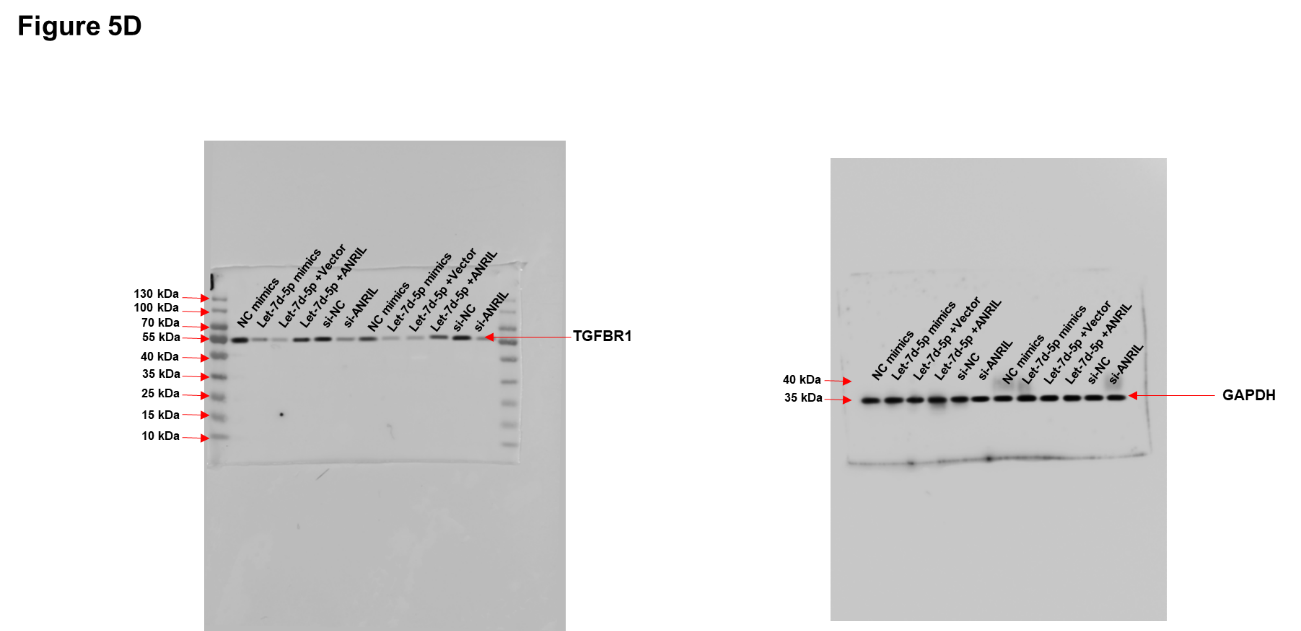


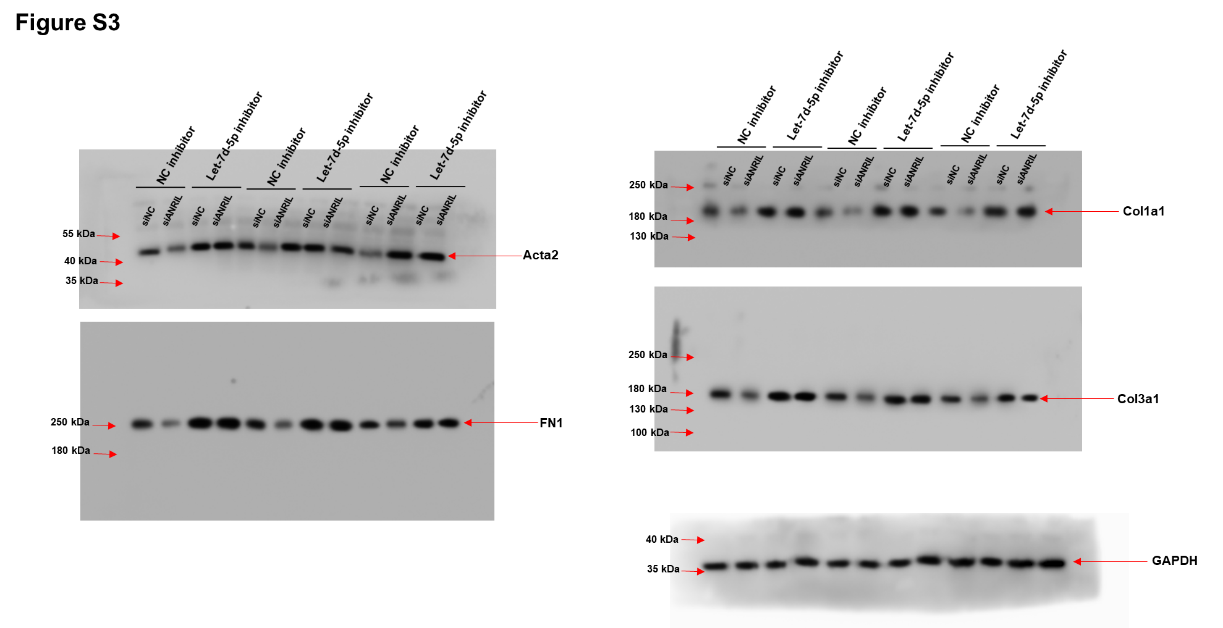


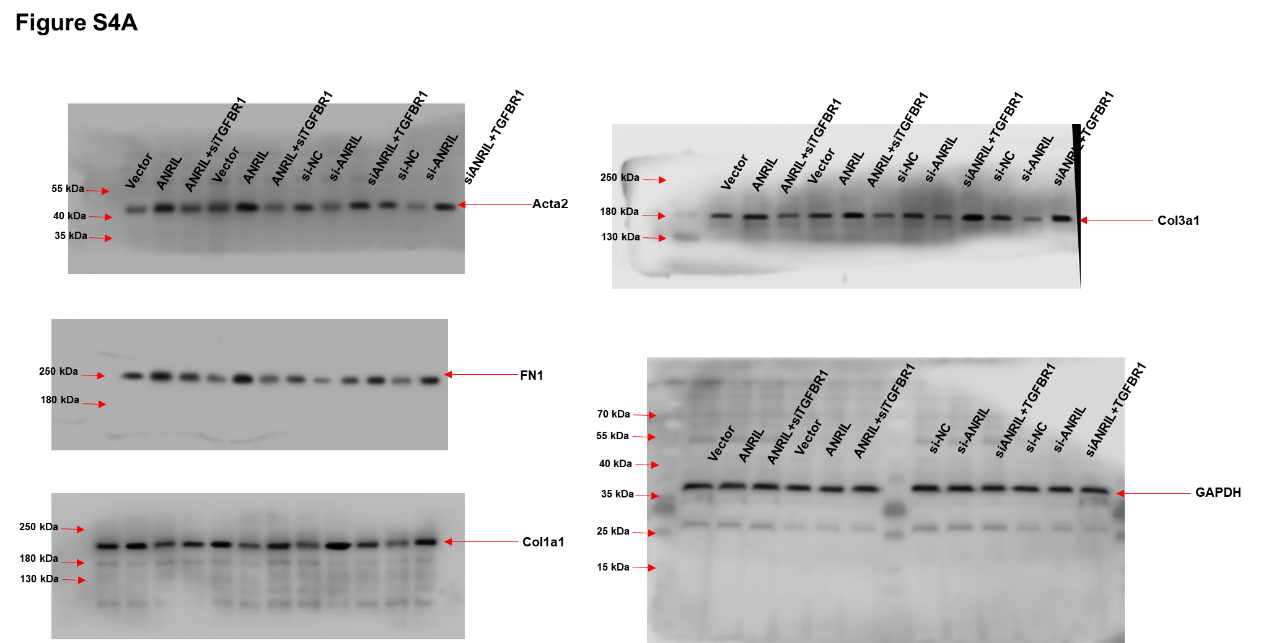

Supplement: original_images_of_western_blot.docx [file KEPI_A_2435682_SM9170.docx]
