# Supplementary material for: ANRIL upregulates TGFBR1 to promote idiopathic pulmonary fibrosis in TGF-β1-treated lung fibroblasts via sequestering let-7d-5p
Source: Epigenetics. 2024 Nov 29;19(1):2435682. doi: 10.1080/15592294.2024.2435682 (PMC11610569; doi:10.1080/15592294.2024.2435682)
Supplement: Supplementary figure.pdf [file KEPI_A_2435682_SM9169.pdf]

Figure S1

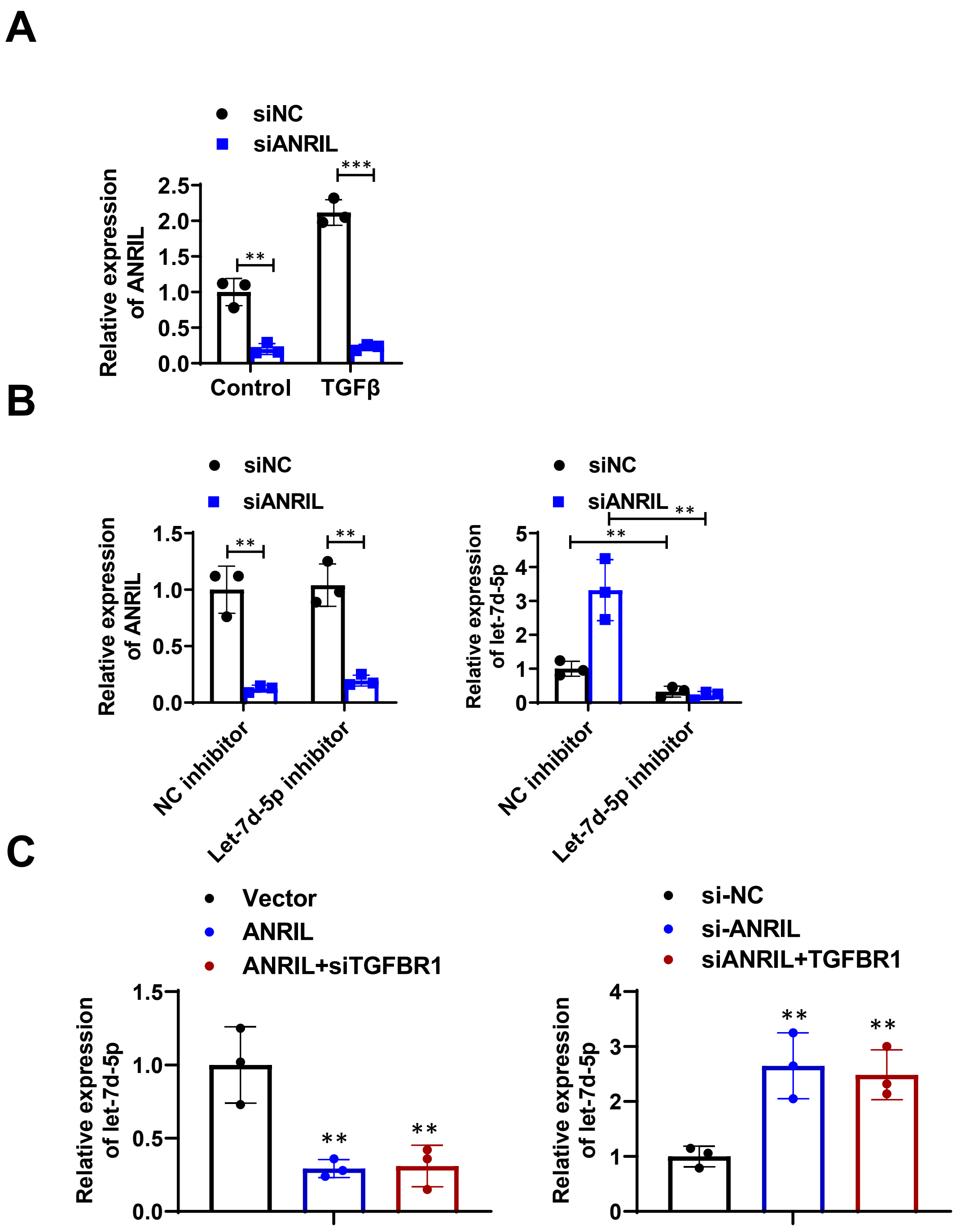

Figure S1. qRT-PCR analysis of the expression of ANRIL and let-7d-5p in the MRC-5 cells. n = 3. Data are presented as mean ± SEM. ***P* < 0.01, ****P* < 0.001.

Figure S2

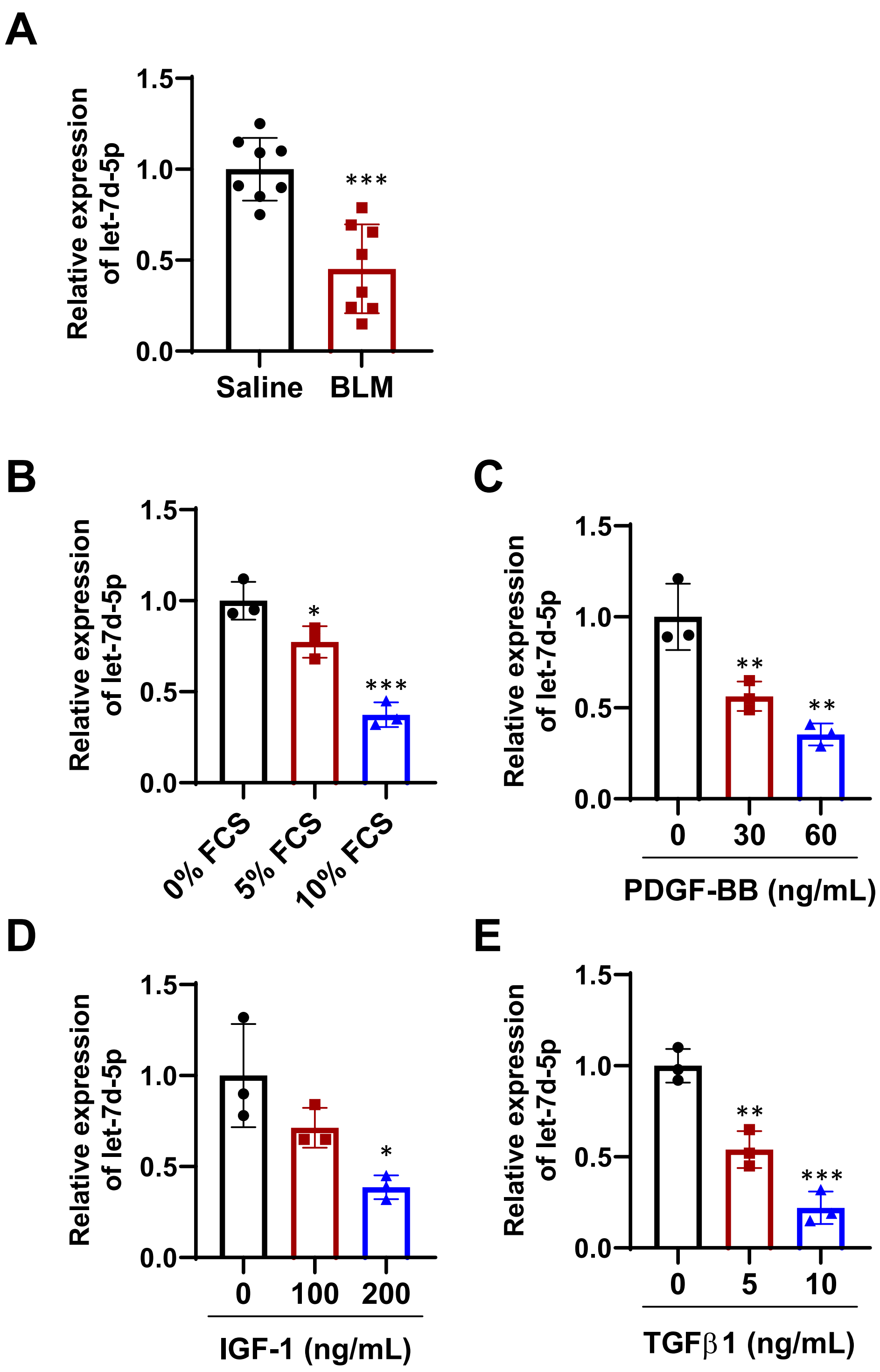

Figure S2. Let-7d-5p was downregulated in IPF lung fibroblasts. A. qRT-PCR analysis of the expression of let-7d-5p in the lungs of BLM-treated mice, n = 6. B-E. MRC-5 cells were stimulated with fetal calf serum (FCS; 2% or 5%), platelet-derived growth factor-BB (PDGF-BB; 30 or 60 ng/ml), insulin-like growth factor 1 (IGF-1; 100 or 200 ng/ml), and transforming growth factor-β1 (TGF-β1; 5 or 10 ng/ml) for 6 h, respectively. let-7d-5p expression was measured by qRT-PCR, n = 3. Data are presented as mean ± SEM; *$P < 0.05$, **$P < 0.01$, ***$P < 0.001$.

Figure S3

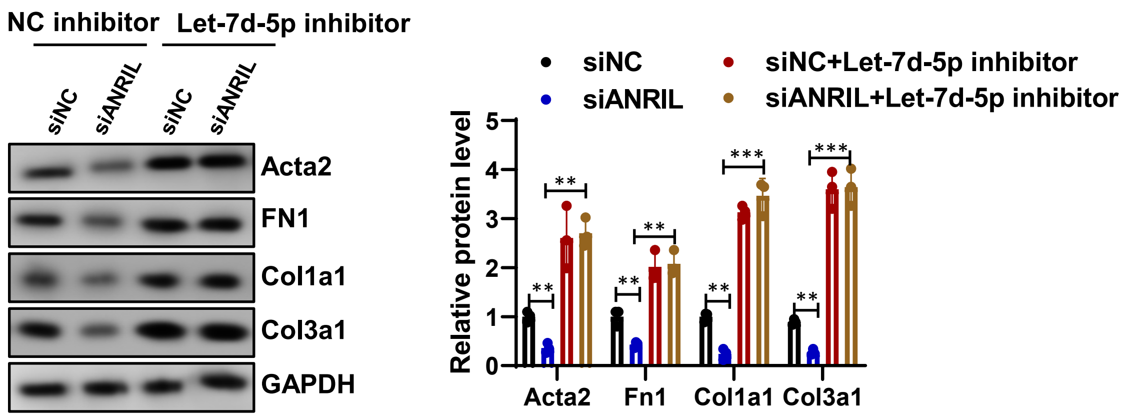

Figure S3. Western blot analysis of the expression of fibrosis-related proteins in TGF-β1-treated lung MRC-5 cells after ANRIL silencing and transfection with let-7d-5p inhibitor. n = 3. Data are presented as mean ± SEM. $**P < 0.01$, $***P < 0.001$.

Figure S4

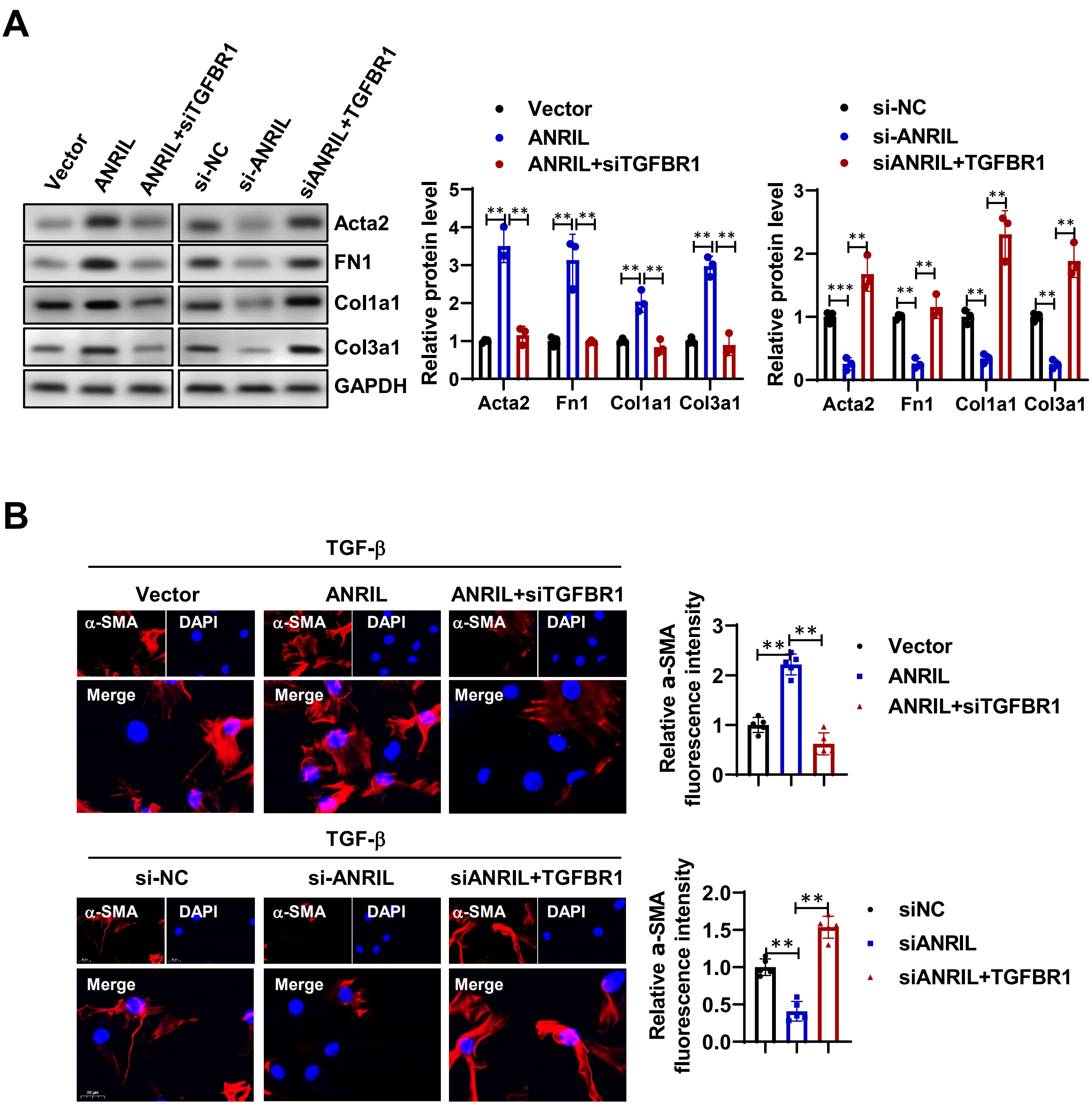

Figure S4. ANRIL promotes lung fibroblasts activation via TGFBR1. A. Western blot analysis of the expression of fibrosis-related proteins in TGF-β1-treated lung MRC-5 cells with indicated treatment. n = 3. B. Immunofluorescence staining indicated that TGF-β1-induced a-SMA positive cells in TGF-β1-treated lung MRC-5 cells with indicated treatment. Scale bar, 50 mm, n = 5. Data are presented as mean ± SEM. ***P* < 0.01.
